# Revisiting Factors Influencing Under-Five Mortality in India: The Application of a Generalised Additive Cox Proportional Hazards Model

**DOI:** 10.3390/ijerph21101303

**Published:** 2024-09-29

**Authors:** Maroof Ahmad Khan, Sumit Kumar Das

**Affiliations:** Department of Biostatistics, All India Institute of Medical Sciences, New Delhi 110029, India

**Keywords:** under-five mortality rate (U5MR), generalised additive models (GAMs), national family health survey (NFHS), generalised additive Cox proportional hazards model

## Abstract

Background: Despite the implementation of various preventive measures, India continues to experience an alarmingly high under-five mortality rate (U5MR). The most recent nationwide data on U5MRs has provided an opportunity to re-examine the associated factors of U5MRs using advanced techniques. This study attempted to identify the associated determinants of U5MRs via the generalised additive Cox proportional hazards method. Methods: This study analysed the fifth round of unit-level data for 213,612 children from the National Family Health Survey (NFHS-5) to identify the risk factors associated with U5MRs, employing a generalised additive Cox proportional hazards regression analysis. Results: The children who had a length of pregnancy of less than 9 months had a 2.621 (95% CI: 2.494, 2.755) times greater hazard of U5MRs than the children who had a gestational period of 9 months or more. The non-linear association with U5MRs was highest in the mother’s age, followed by the mother’s haemoglobin, the mother’s education, and household wealth score. The relationships between the mother’s age and the mother’s haemoglobin level with the U5MR were found to be U-shaped. Conclusions: This study highlights the importance of addressing maternal and socioeconomic factors while improving access to healthcare services in order to reduce U5MRs in India. Furthermore, the findings underscore the necessity for more sophisticated approaches to healthcare delivery that consider the non-linear relationships between predictor variables and U5MRs.

## 1. Introduction

The under-five mortality rate (U5MR) is a key metric for measuring the health and well-being of a society. Since 1990, the global U5MR has dropped by 59%, from 93 deaths per 1000 live births in 1990 to 38 in 2021 [1]. Sustainable Development Goal (SDG) 3.2 aims to reduce the U5MR to 25 by 2030. As of 2019, 122 out of 195 nations had attained the U5MR target set by the SDG, and 20 countries were advancing towards achieving it by 2030. However, 53 countries must accelerate their efforts to meet the target by 2030. In 2019, Sub-Saharan Africa had the world’s highest U5MR, at approximately 75.8 deaths per 1000 live births. The high U5MR is heavily concentrated in African and South Asian countries, and the combined U5MR from Nigeria and India constitutes almost a third of the total [2].

India’s current U5MR is still alarmingly high despite significant precautions in the last few decades. Following the 1978 Alma Ata declaration, India initiated various actions to reduce the U5MR, including the launch of the National Diarrhoeal Disease Control Programme in the same year. Although India could not achieve the Millennium Development Goal (MDG-4) by 2015, the U5MR in India has dropped from 109 deaths in 1992–1993 to 41 deaths in 2019–2021 [3]. Keeping a more ambitious target than SDG 3.2, India’s new National Health Policy (NHP) aims to reduce the U5MR to 23 by 2025. Nonetheless, expecting India to reach global and national targets with the current rate of decline seems improbable. Despite implementing extensive policies and programmes, India’s current rate of decline in U5MRs calls for a fresh investigation to identify more vulnerable segments of the population facing a higher risk of U5MRs using the latest dataset and a different methodological approach. 

The U5MR within countries varies across sub-populations in India. This variation is particularly linked to socioeconomic and regional factors [4]. According to recent nationally representative data NFHS-5 (2019–2021), the U5MR was highest among the scheduled caste group (48.9). The poorest quintile’s mortality (59) was about thrice as high as the richest quintile’s (20.1). Mothers with no schooling had nearly 2.5 times higher U5MRs than those educated for 12+ years. Rural areas had a higher rate (45.7) than urban areas (31.5). A mother’s age of less than 20 years (52.5) and at or more than 40 years of childbirth (72.9) were associated with higher U5MRs [3]. Several attempts have been made to explore the sub-groups or regions that are at higher risk of U5MRs using previously available datasets and conventional methods [4,5,6,7,8,9,10,11,12,13,14,15,16,17]. The place of residence [13], wealth status [7], religion [9], type of household [10], type of cooking fuel [11], the mother’s age at birth [5,18], the mother’s education [8], the mother’s haemoglobin level [19], birth order [12], caesarean section delivery [6], length of pregnancy [20], wanted pregnancy [14], place of delivery [15], whether the mother consumes tobacco [16], and the sex of the child [17] are the common risk factors explored in the previous study. The existing situation of U5MRs in India underscores the need to re-evaluate the associated risk factors with advanced methods, and this opportunity is facilitated by the recent release of national-level data on U5MRs. Hence, in this study, we attempted to reassess the risk factors for U5MRs using a generalised additive Cox proportional hazards model. This statistical technique works with time-to-event data and incorporates the methods of generalised additive models for flexible modelling. Unlike traditional Cox proportional hazards models, it does not rely on proportional assumptions. This model employs smoothing functions to fit continuous data effectively [21].

## 2. Materials and Methods

### 2.1. Data and Sample Design

This study used the fifth round of unit-level data from the National Family Health Survey (NFHS -5) of 2019–2021. The data are freely accessible at the official website of the Demographic Health Survey (DHS). All the survey protocols were validated by the International Institute for Population Sciences (IIPS), which is the nodal organisation. One of the largest demographic and health surveys, NFHS-5, has been conducted in 707 districts across India. The sample size of NFHS-5 consists of 101,839 males and 724,115 females from 636,699 Indian households. The surveys gathered essential information on sociodemographic traits, marriage, fertility, childbirth, immunisations, nutrition, contraception, fertility preference, sexual behaviour, views towards gender roles, HIV/AIDS, and anthropometric measures. Census villages and urban blocks serve as the first-stage units for rural and urban regions, respectively; in the two-stage stratified probability proportional to size, the sample design used NFHS-5, while households served as the second-stage unit [22]. The survey collected information from 232,920 kids aged 0 to 59 months who were born five years before the date of the interview of women aged 15 to 49 years. In total, 213,612 kids were considered the analytical sample for this study. Figure S1 contains the flowchart for the analytical sample for this study.

### 2.2. Outcome Variable

The outcome of interest is the survival status of children within the age groups of 0 to 59 months. Children who passed away within five years of birth were assigned the code 1. Children who lived for 59 months or longer were categorised as 0. In NFHS-5, data on child survival were retrospectively gathered by interviewing the mother.

### 2.3. Explanatory Variables

The explanatory variables included in this study are broadly classified as household characteristics, mother’s characteristics, and children’s characteristics. The household characteristics include place of residence (rural/urban), religion (Hindu/other), wealth status of a household (household wealth score available in the NFHS-5 data [3]), type of cooking fuel (smoke/no smoke), and household structure (nuclear family/non-nuclear family). Nuclear households are composed of a married couple or a man or woman living alone or with unmarried children (biological, adopted, or fostered) with or without unrelated individuals [3]. The mother’s characteristics include her current age in years, haemoglobin level (g/dL) of a mother adjusted for altitude and smoking, mother’s education in single years, and tobacco consumption (yes/no). The children’s characteristics include birth order (one, two, three, or more), caesarean section delivery (yes/no), length of pregnancy (less than nine months/nine months or more), wanted pregnancy (yes/no), and place of delivery (home/institution).

### 2.4. Statistical Analysis

For categorical data, the variables included in this study were summarised using frequency and weighted percentage distribution. For continuous data, median and interquartile ranges were reported.

The outcome of this study was time-to-event data. The age of the child in months at the time of interview was considered ‘time’. The survival status of the child was considered an event variable, where the death of a child was considered an ‘event’. The most popular method used to fit this type of data is the Cox proportional hazards model [23]. Univariate and multivariable Cox proportional hazards models were initially applied, and assumptions were checked to assess the applicability of the model to the data. The results of conventional Cox proportional hazards regression analysis are presented in Table S1.

The generalised additive Cox proportional hazards model, a type of generalised additive model (GAM), provides a flexible, non-parametric alternative to the Cox proportional hazards regression model. By substituting an additive predictor for a linear predictor, it combines the non-linearity of spline regression approach with the linearity of traditional linear regression techniques. GAMs automatically determine the optimal level of non-linearity in the data and handles covariates with numerous predictors efficiently compared to other non-linear regression methods [24].

The mathematical form of a GAM can be written as

(1) $$h(t|X,Z)=h_{0}\left(t\right)(\exp\left(\beta_{1}X+\sum_{j=1}^{m}\beta_{j}z_{j}+\sum_{j=m+1}^{p}S_{j}(z_{j})\right))$$

where h(t|X,Z) is the hazard function at time t, with X representing the variables of interest and $Z=z_{1}+z_{2}+\ldots +z_{p}$ representing the list of covariates. $h_{0}\left(t\right)$ is the baseline hazard for the reference subjects with all covariates equal to 0. The βs for categorical variables are estimated using penalised likelihood, and the likelihood is maximised using the Newton–Raphson iterative method [25]. The $S_{j}$’s (j=(m+1) to p) are the functions estimated using penalised regression spline smoother in backfitting method [26,27]. The findings of the GAM obtained from continuous predictors are expressed as a series of partial residual plots. These plots illustrate the relationship between the smoothed predictors and the dependent variable rather than as a single regression parameter.

The data preparation and statistical analyses were performed using STATA 18.0 (College Station, TX, USA) and RStudio software 2022.12.0 software. As the data used in this study have a complex survey design, the sampling weights were adjusted to obtain the percentages.

## 3. Results

A total of 213,612 children were included in this study. Of the total children, 73.75% were from rural areas. The religious composition of the study shows that 79.59% belonged to Hinduism and 20.41% of children belonged to other religious categories. Among the other religious categories, 52.86% were Muslim, 31.83% were Christian, 6.38% were Sikh, and 3.80% were Buddhist/Neo-Buddhist. The average age of mothers was 27 years, and mothers had an average of 8 years of education. The average haemoglobin level among mothers was 11.5 g/decilitre. Of the total surveyed children, 33.81% were first-order births, and 28.15% of them were third-order or higher births. Around twenty-one percent of children were delivered by caesarean section, and almost thirteen percent of children had a pregnancy duration of less than nine months. Almost three and a half percent of the children’s mothers had a history of tobacco consumption. Almost fifty-two percent of the children were male (Table 1).

Table 2 shows the descriptive statistics of the survival status of children based on their background characteristics. The percentage of deceased children was higher in rural areas compared to urban areas. Households that used smokeless cooking fuel had a lower percentage of deceased children. The U5MR was lowest for the second-born children (2.97%). Around 5.72% of children born at home did not survive until five years of age. Children whose mothers had a history of tobacco consumption did not survive in approximately 5.17% of cases. Male children had a higher percentage of mortality (3.99%) than female children (3.47%).

Table 3 presents the adjusted and unadjusted hazard ratios for the risk factors related to U5MRs from the generalised additive Cox proportional hazards model. Children from rural areas had a 1.41 times higher risk of experiencing U5MRs than those from urban areas. However, the effect became insignificant after adjusting for other factors. Children belonging to religions other than Hinduism had a lower hazard (adjusted hazard ratio (AHR) = 0.778 (95% CI: 0.737, 0.821), *p* < 0.001) of U5MRs than children belonging to Hinduism. Children who had a gestational period of less than nine months had 2.621 times higher hazard of U5MRs than the children who had gestational periods of nine months or more (*p* < 0.05). Children from unplanned pregnancies had a higher hazard (AHR = 1.274 (95% CI: 1.179, 1.376), *p* < 0.001) of U5MRs than others. Mothers with a history of tobacco consumption had a higher hazard for child mortality (AHR = 1.108 (95% CI: 1.02, 1.204), *p* < 0.05). Even after controlling for other risk factors, female children had a lower hazard (AHR = 0.855 (95% CI: 0.818, 0.893), *p* < 0.001) of U5MR than male children.

Table 4 presents the estimated degrees of freedom (EDF), reference degrees of freedom (RDF), $\chi^{2}$ values, *p*-values, and percentage of deviance explained obtained from unadjusted and adjusted generalised additive Cox proportional hazards models. The EDF is a measure that indicates the flexibility of a smooth term in fitting data. An EDF of 1 signifies linearity, while an EDF greater than 1 indicates non-linearity. The RDF is the maximum EDF that can be achieved for a smooth term. An EDF close to the RDF suggests that the smooth term captures the underlying non-linear relationship between predictor and response variables. A significantly greater EDF than 1 indicates a non-linear and more flexible term. Comparing EDF values across models or variables helps identify stronger non-linear relationships with the response variable. The results showed statistically significant non-linearity in all four variables, with the mother’s age exhibiting the highest non-linearity, followed by the mother’s haemoglobin, education, and wealth score.

**Figure 1:**
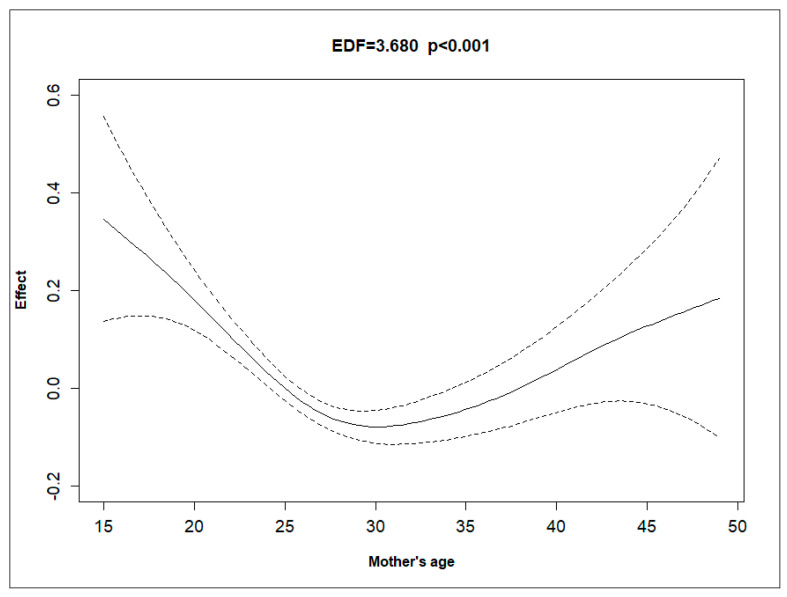
Non-linear effect of mother’s age (in years) on the hazard rate, with 95% confidence intervals, showing a significant relationship (EDF = 3.680, *p* < 0.001).

**Figure 2:**
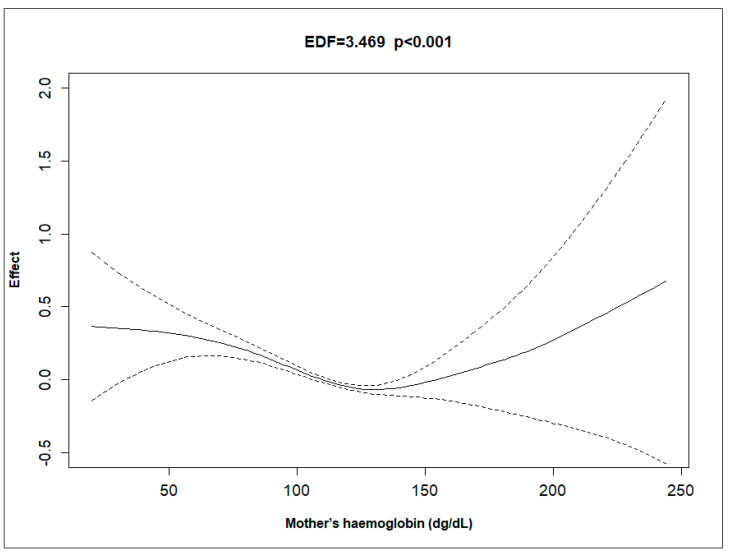
Non-linear effect of mother’s haemoglobin (dg/dL) on the hazard rate, with 95% confidence intervals, showing a significant relationship (EDF = 3.469, *p* < 0.001).

**Figure 3:**
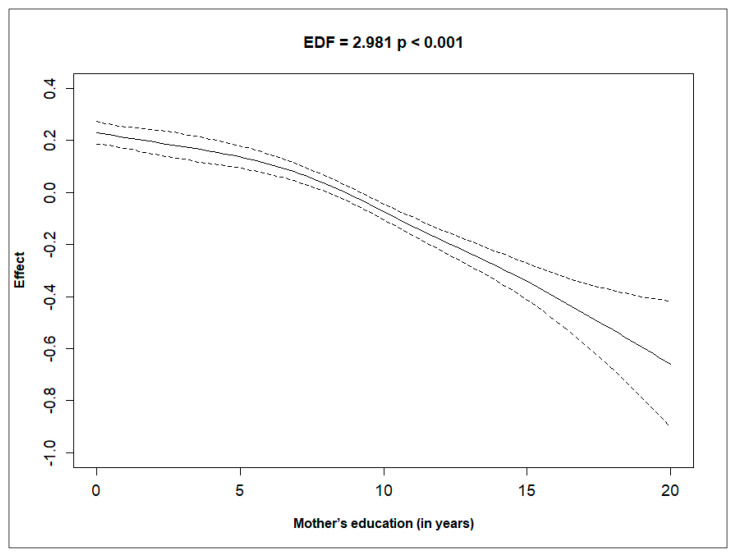
Non-linear effect of mother’s education (in years) on the hazard rate, with 95% confidence intervals, showing a significant relationship (EDF = 2.981, *p* < 0.001).

**Figure 4:**
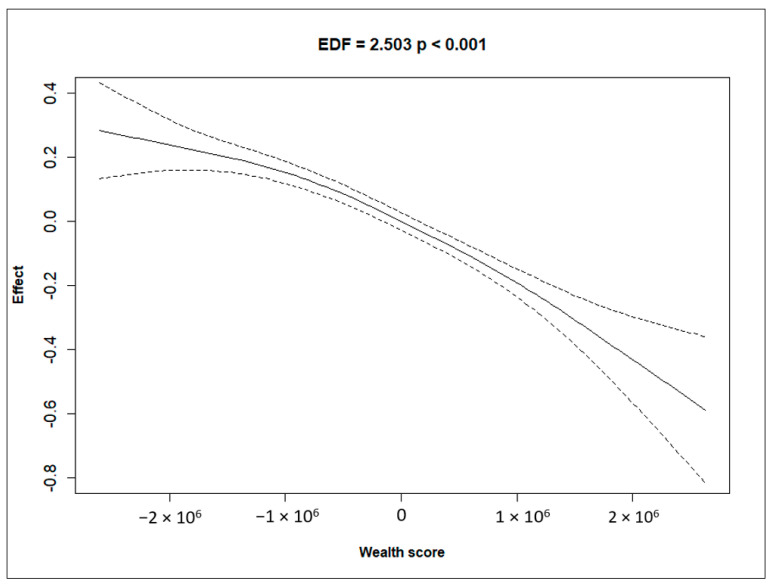
Non-linear effect of wealth score on the hazard rate, with 95% confidence intervals, showing a significant relationship (EDF = 2.503, *p* < 0.001).

Figure 1, Figure 2, Figure 3 and Figure 4 depict the effect of U5MRs across ranges of mothers’ age, mothers’ education, mothers’ haemoglobin level, and wealth score of households. The relationship between the mother’s age and U5MR was found to be U-shaped; children born to mothers who were either very young (<23 years) or older (>32 years) had a higher risk of U5MRs (Figure 1). Likewise, the mother’s haemoglobin level showed a concave upward shape, where the U5MRs were found to be low, between approximately 11.5 and 14 gm/dL haemoglobin levels (Figure 2). The pattern of U5MRs across mother’s education and wealth score showed a similar pattern, i.e., with lower values, the mortality is higher, and it diminishes with a higher level of education (Figure 3) and wealth score, respectively (Figure 4).

## 4. Discussion

This study is unique in two aspects: first, it re-examined the risk factors for U5MRs using the most recent available data; second, the application of the generalised additive Cox proportional hazards model is unique in identifying the risk factors for U5MRs in the Indian context. The major findings of this study are as follows: preterm birth emerged as the most significant risk factor for U5MRs. Second, children born to mothers who fell outside the age range of 23 to 32 years were found to have an elevated risk of mortality before reaching the age of five. Third, higher maternal education was associated with a decrease in U5MRs. Fourth, a higher wealth score was linked to a reduction in U5MRs.

Our study found that birth before 37 weeks of gestation was associated with an increased risk of U5MRs. Premature babies face numerous challenges due to underdeveloped organs and body systems. These make them more susceptible to infections, respiratory distress, and other health complications that can lead to mortality [28]. Moreover, specialised medical care and interventions may not be universally available or affordable for premature babies, and premature birth itself can be associated with maternal health issues that further increase the risk of mortality for both the mother and baby [29]. The under-five mortalities in 2019 were approximately 5.30 million cases, with preterm birth complications accounting for a significant portion of these fatalities (17.7% approximately) [20]. The persistent higher risk of mortalities in premature babies highlights the importance of improving access to prenatal care and specialised medical interventions for preterm infants, as well as addressing the underlying causes of preterm birth.

The U-shaped relationship between maternal age and U5MRs underscores the importance of adopting a lifecycle approach to maternal and child health and addressing the complex interplay between maternal and child health outcomes. This finding aligns with other previous studies by Finlay et al. [5] and Meitei et al. [6].

This study revealed a U-shaped relationship between maternal haemoglobin levels and U5MRs, indicating that both low and high levels are associated with increased risk. Low haemoglobin (anaemia) can lead to various health issues affecting maternal and child outcomes [19]. High haemoglobin may indicate conditions such as polycythaemia, hypertension, and blood clots, which also pose health risks [30]. Monitoring and appropriate interventions, like iron supplementation for anaemia, are crucial during pregnancy. Additionally, interventions to prevent and treat high haemoglobin, such as improving hydration and oxygenation, are important for improving maternal and child health outcomes.

We also found that higher wealth scores were associated with lower U5MR. This is because families with higher wealth scores may have better access to healthcare facilities and services, immunisation programs, improved living conditions, clean water, sanitation, and nutritious food. Improving economic conditions and reducing poverty can lead to better child health outcomes. In addition to this, policies should further focus on reducing inequalities and enhancing access to essential healthcare and services to reduce U5MRs [31].

Similar to findings from previous studies [7,8], this study suggests that higher maternal education is linked to lower U5MRs, emphasising its role in improving child health outcomes. Maternal education may enhance informed decision making regarding healthcare, healthy behaviours, and illness management for both the mother and child. Access to health information and resources is more likely for mothers with higher education levels, enabling better health-related decisions for themselves and their children.

Previous studies on U5MR risk factors in India have informed targeted interventions by policymakers, primarily assuming linear relationships between predictors and U5MRs in their statistical methods [6,8,32,33]. However, the relationship between predictors and U5MRs was not always linear. For example, maternal ages were found to have shown a “U-shaped” or “elongated L-shaped” relationship with U5MRs [34]. The existence of these specific patterns in the data necessitates a different method to address the associated risk factors. The generalised additive Cox proportional hazards model played an important role in modelling the relationship between the response variable and predictor variables as the sum of multiple non-linear functions [24]. This study extends beyond a mere re-examination of the impact of a mother’s age, encompassing its non-linear aspects. Additionally, we undertake a fresh evaluation of the non-linear relationships associated with the wealth quintile, mother’s haemoglobin level, mother’s education, and household wealth scores.

This study found a modest J-shaped or mildly U-shaped relationship between birth order and U5MRs. After adjusting for other risk factors, the hazard ratios of U5MRs among firstborn children and those with three or more siblings were higher as compared to second-birth-order children. This aligns with the study, which also identified a modest J-shaped relationship between birth order and infant mortalities [35]. First- and last-order children are comparatively at a higher risk of mortality than those born in the middle. Firstborn children are more vulnerable to the risk of U5MR because they are born to parents who have less parenting experience, whereas children of a higher birth order face increased risk as the family size increases, leading to the depletion of both economic and emotional paternal resources. This particularly disadvantages the youngest children [35].

The findings of this study have important implications for policymakers and healthcare practitioners working to reduce U5MRs in India. The results of this study suggest that interventions targeted at promoting girl child education, increasing institutional delivery, reducing unwanted pregnancies, ensuring deliveries at 37 weeks of gestation, and addressing inequalities in access to health care services across different economic strata have the greatest impact on reducing U5MRs. Additionally, the findings of this study highlight the need for more nuanced approaches to healthcare delivery that take into account the non-linear relationships between predictor variables and U5MRs. This non-linear relationship between predictors and U5MRs offers an updated, detailed understanding for policymakers and healthcare professionals to effectively target interventions and reduce U5MRs. Overall, this study demonstrates the value of using a generalised additive Cox proportional hazards model to identify non-linear relationships between predictor variables and U5MRs. It also provides important insights for improving child health outcomes in India.

This study has some limitations. First, the survey data on child survival was obtained through interviews, considering a five-year recall period with women aged between 15 and 49, which forms the basis of the analysis of this study. Due to the retrospective nature of the data, there is a potential for recall bias when mothers report the age at death of their children. Accurate age-at-death information for each child is essential in order to calculate the actual proportion of fatalities in a given age group and to obtain an accurate overall estimate of mortality [36]. Second, because of the substantial number of missing cases, we were unable to incorporate birth weight and preceding birth interval into the regression analysis. These risk factors were found to be statistically significant factors in the previous study [33]. Finally, antenatal care information was only gathered in relation to the most recent delivery within the five years preceding the date of the survey. As a result, we were unable to include factors linked to antenatal care in the regression models.

## 5. Conclusions

This study extends the existing evidence by examining U5MR risk factors in India using the fifth round of NFHS data from approximately 0.21 million births. Advanced statistical models, such as the generalised additive Cox proportional hazards model, enabled us to uncover the non-linear effects of risk factors, an aspect often overlooked in the literature. We effectively demonstrated the impact of variables such as maternal age, household wealth, education, and haemoglobin levels on newborn mortality. Additionally, we highlighted changes in the associations of variables like child sex and religion with U5MRs compared to the previous survey. This study underscores the significance of addressing maternal and socioeconomic factors and improving healthcare access to reduce U5MRs in India.

## Figures and Tables

**Table 1:** Description of study characteristics and weighted percentage distribution of the under-five children in India, 2019–2021.

Variables	Frequency	Weighted Percentage
**Child survival status**		
Alive	205,657	96.26
Death	7955	3.74
**Place of residence**		
Urban	42,761	26.25
Rural	170,851	73.75
**Wealth score ^$^**	−156,481 (−924,340, 651,000)	
**Religion**		
Hindu	156,265	79.59
Others	57,347	20.41
**Type of household**		
Nuclear	83,135	37.21
Non-Nuclear	130,477	62.79
**Type of cooking fuel**		
No Smoke	95,933	50.05
Smoke	117,679	49.95
**Mother’s age (in years) ^$^**	27 (24, 30)	
**Mother’s education (in years) ^$^**	8 (3, 11)	
**Haemoglobin level (gm/dL) ^$^**	11.5 (10.5, 12.5)	
**Birth order**		
One	70,460	33.81
Two	79,801	38.04
Three or more	63,351	28.15
**Caesarean section**		
No	173,413	78.87
Yes	40,199	21.13
**Length of pregnancy (in months)**		
More than 9 months	187,147	87.21
Less than 9 months	26,465	12.79
**Wanted pregnancy**		
Yes	199,110	92.77
Later/No	14,502	7.23
**Place of delivery**		
Institution	184,044	88.64
Home	29,568	11.36
**Mother consumes tobacco?**		
No	199,280	96.56
Yes	14,332	3.44
**Sex of child**		
Male	110,603	51.87
Female	103,009	48.13
Total	213,612	100

**^$^** Median and interquartile range (IQR) are reported.

**Table 2:** Description of study characteristics and weighted percentage distribution of under-five children by survival status in India, 2019–2021.

Variables	Frequency	Weighted Percentage	Frequency	Weighted Percentage
	Survival		Deceased	
**Place of Residence**				
Urban	41,554	96.01	1207	3.99
Rural	164,103	96.53	6748	3.47
**Wealth Score ^$^**	−142,360 (−912,320, 663,199)		−509,630 (−1,146,890, 278,940)	
**Religion**				
Hindu	150,120	96.18	6145	3.82
Others	55,537	96.58	1810	3.42
**Type of Household**				
Nuclear	79,861	95.89	3274	4.11
Non-Nuclear	125,796	96.48	4681	3.52
**Type of cooking fuel**				
No Smoke	93,165	97.09	2768	2.91
Smoke	112,492	95.43	5187	4.57
**Mother’s age (in years) ^$^**	27 (24, 30)		26 (23, 30)	
**Mother’s education (in years) ^$^**	8 (3, 11)		7 (0, 10)	
**Haemoglobin level (gm/dL) ^$^**	11.5 (10.5, 12.5)		11.3 (10.3, 12.3)	
**Birth order**				
One	76,860	96.35	2941	3.65
Two	68,302	97.03	2158	2.97
Three or more	60,495	95.22	2856	4.78
**Caesarean Section**				
No	166,578	95.98	6835	4.02
Yes	39,079	97.29	1120	2.71
**Length of pregnancy (in months)**				
More than 9 months	181,311	96.87	5836	3.13
Less than 9 months	24,346	92.08	2119	7.92
**Wanted Pregnancy**				
Yes	191,877	96.34	7233	3.66
Later/No	13,780	95.19	722	4.81
**Place of delivery**				
Institution	177,644	96.51	6400	3.49
Home	28,013	94.28	1555	5.72
**Mother consumes tobacco?**				
No	191,973	96.31	7307	3.69
Yes	13,684	94.83	648	5.17
**Sex of child**				
Male	106,191	96.01	4412	3.99
Female	99,466	96.53	3543	3.47
Total	205,657		7955	

**^$^** Median and interquartile range (IQR) are reported.

**Table 3:** Unadjusted and adjusted hazard ratios (HRs) of the risk factors and corresponding *p*-values of under-five mortalities obtained from generalised additive Cox proportional hazards model in India, 2019–2021.

Variables	Unadjusted HR (95% CI)	*p*-Value	Adjusted HR (95% CI)	*p*-Value
**Place of Residence**				
Urban	Ref.		Ref.	
Rural	1.41 (1.326, 1.499)	<0.001	0.974 (0.909, 1.044)	0.4565
**Religion**				
Hindu	Ref.		Ref.	
Others	0.799 (0.759, 0.842)	<0.001	0.778 (0.737, 0.821)	<0.001
**Type of Household**				
Nuclear	Ref.		Ref.	
Non-Nuclear	0.928 (0.888, 0.971)	0.0011	1.049 (0.999, 1.101)	0.0565
**Type of cooking fuel**				
No Smoke	Ref.		Ref.	
Smoke	1.538 (1.469, 1.611)	<0.001	1.075 (1.013, 1.141)	0.0165
**Birth order**				
One	Ref.		Ref.	
Two	0.828 (0.783, 0.875)	<0.001	0.817 (0.77, 0.867)	<0.001
Three or more	1.222 (1.161, 1.287)	<0.001	1.015 (0.948, 1.086)	0.6738
**Caesarean Section**				
No	Ref.		Ref.	
Yes	0.71 (0.666, 0.756)	<0.001	0.976 (0.912, 1.044)	0.4771
**Length of pregnancy (in months)**				
More than 9 months	Ref.		Ref.	
Less than 9 months	2.614 (2.487, 2.747)	<0.001	2.621 (2.494, 2.755)	<0.001
**Wanted Pregnancy**				
Yes	Ref.		Ref.	
Later/No	1.393 (1.29, 1.503)	<0.001	1.274 (1.179, 1.376)	<0.001
**Place pf delivery**				
Institution	Ref.		Ref.	
Home	1.502 (1.421, 1.588)	<0.001	1.216 (1.146, 1.291)	<0.001
**Mother consumes tobacco?**				
No	Ref.		Ref.	
Yes	1.224 (1.13, 1.326)	<0.001	1.108 (1.02, 1.204)	0.0156
**Sex of child**				
Male	Ref.		Ref.	
Female	0.861 (0.824, 0.9)	<0.001	0.855 (0.818, 0.893)	<0.001

**Table 4:** Results of hypotheses tests of the fitted generalised additive Cox proportional hazards model.

	Smooth Terms	Wealth Score	Mother’s Age	Mother’s Education	Mother’s Haemoglobin
Unadjusted	EDF ^^^	3.044	4.435	5.452	3.915
	RDF ^$^	3.83	5.424	6.466	4.836
	$\chi^{2}$-values	629.5	126.2	639.6	121.6
	p-values	<0.001	<0.001	<0.001	<0.001
	Deviance Explained	1.16%	0.15%	1.24%	0.20%
Adjusted	EDF ^^^	2.503	3.68	2.981	3.469
	RDF ^$^	3.173	4.581	3.662	4.352
	$\chi^{2}$-values	61.36	44.6	159.47	55.36
	p-values	<0.001	<0.001	<0.001	<0.001
	Deviance Explained		4.29%		

^^^ EDF: estimated degrees of freedom; ^$^ RDF: reference degrees of freedom.

## Data Availability

This study used an anonymous publicly available Demographic and Health Survey (DHS) dataset freely available on a data repository: https://dhsprogram.com/data/dataset/India_Standard-DHS_2020.cfm?flag=1 (accessed on 10 April 2024).

## Supplementary Materials

The following supporting information can be downloaded at: https://www.mdpi.com/article/10.3390/ijerph21101303/s1, Figure S1: Flowchart for effective number of samples from NFHS 5 (2019–2021). Table S1: Unadjusted and adjusted hazard ratios obtained from conventional univariate Cox proportional hazards model.
