# Supplementary material for: Revisiting Factors Influencing Under-Five Mortality in India: The Application of a Generalised Additive Cox Proportional Hazards Model
Source: Int J Environ Res Public Health. 2024 Sep 29;21(10):1303. doi: 10.3390/ijerph21101303 (PMC11507100; doi:10.3390/ijerph21101303)
Supplement: Supplementary file 1 [file ijerph-21-01303-s001.zip › ijerph-3195466-supplementary.pdf]

Figure S1: Flowchart for effective number of samples from NFHS-5 (2019 - 21)

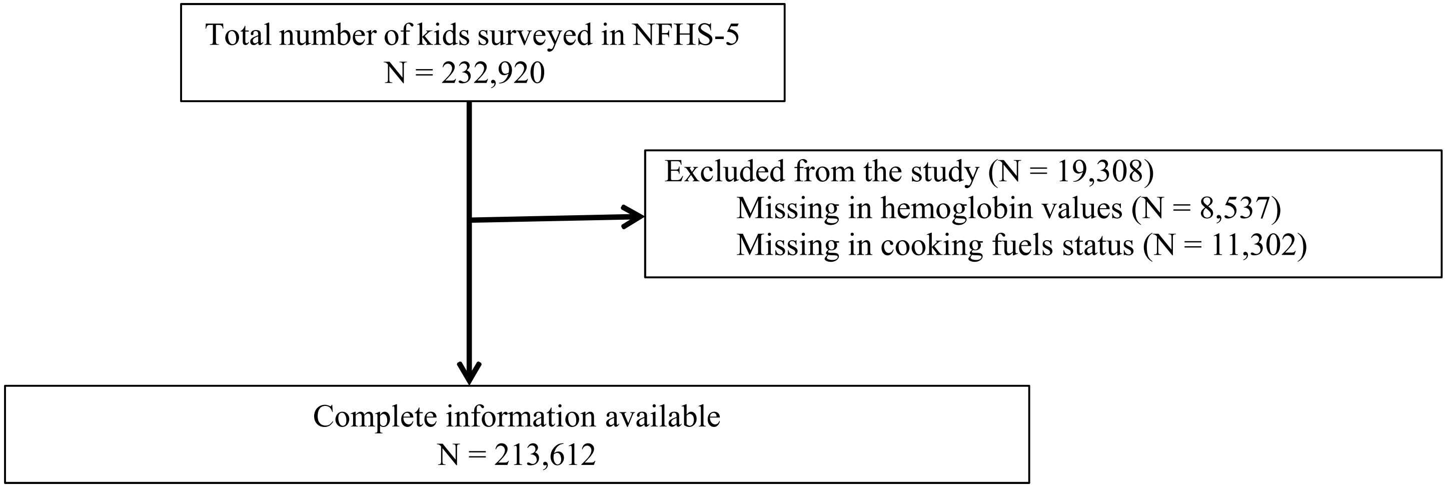

Table S1: Unadjusted and adjusted hazard ratios obtained from conventional univariate cox proportional hazard model.

Variables	UHR*(95% CI)	p-value	PH assumption^	Influential Observation	linearity	AHR[#](95% CI)	p-value
**Place of Residence**							
Urban							
Rural	1.43(1.326,1.499)	<0.001	Retained	Not present	Retained	-	-
**Wealth Score**	0.999(0.999,0.999)	<0.001	**Violated**	Not present	Retained	0.999(0.999,0.999)	<0.001
**Religion**							
Hindu	Ref.					Ref.	
Others	0.799(0.759,0.842)	<0.001	**Violated**	Not present	Retained	0.795(0.753,0.839)	<0.001
**Type of Household**							
Nuclear	Ref.					Ref.	
Non-Nuclear	0.928(0.888,0.971)	0.001	**Violated**	Not present	Retained	1.054(1.00,1.106)	0.032
**Type of cooking fuel**							
No Smoke						1.079(1.018,1.145)	0.010
Smoke	1.538(1.469,1.611)	<0.001	Retained	Not present	Retained		
**Mother's age (in years)**	0.992(0.988,0.997)	0.001	**Violated**	Not present	**Violated**	0.99(0.985,0.994)	<0.001
**Mother's education (in years)**	0.945(0.941,0.949)	<0.001	**Violated**	Not present	**Violated**	0.965(0.96,0.97)	<0.001
**Haemoglobin level (gm/dl)**	0.993(0.992,0.994)	<0.001	Retained	**Present**	**Violated**	0.995(0.994,0.997)	<0.001
**Birth order**							
One	Ref.					Ref.	
Two	0.828(0.783,0.875)	<0.001				0.799(0.76,0.84)	<0.001
Three or more	1.222(1.161,1.287)	<0.001	**Violated**	Not present	Retained	-	-
**Caesarean Section**							
No	Ref.						
Yes	0.71(0.666,0.756)	<0.001	**Violated**	Not present	Retained	-	-
**Length of pregnancy (in months)**							
More than 9 months	Ref.					Ref.	
Less than 9 months	2.614(2.487,2.747)	<0.001	**Violated**	Not present	Retained	2.621(2.494,2.755)	<0.001
**Wanted Pregnancy**							
Yes	Ref.					Ref.	
Later/No	1.393(1.29,1.503)	<0.001	Retained	Not present	Retained	1.291(1.196,1.394)	<0.001
**Place of delivery**						Ref.	
Institution	Ref.						
Home	1.502(1.421,1.588)	<0.001	**Violated**	Not present	Retained	1.215(1.145,1.289)	<0.001
**Mother consumes tobacco?**							
No	Ref.					Ref.	
Yes	1.224(1.13,1.326)	<0.001	**Violated**	Not present	Retained	1.11(1.022,1.206)	0.013
**Sex of child**							
Male	Ref.					Ref.	
Female	0.861(0.824,0.9)	<0.001	**Violated**	Not present	Retained	0.855(0.818,0.894)	<0.001

*UHR: Unadjusted hazard ratio; [#]AHR: Adjusted hazard ratio; ^Proportionality hazard assumption
